# Effect of surgical parameters on the biomechanical behaviour of bicondylar total knee endoprostheses – A robot-assisted test method based on a musculoskeletal model

**DOI:** 10.1038/s41598-019-50399-3

**Published:** 2019-10-10

**Authors:** M. Kebbach, R. Grawe, A. Geier, E. Winter, P. Bergschmidt, D. Kluess, D. D’Lima, C. Woernle, R. Bader

**Affiliations:** 1 Department of Orthopaedics, University Medicine Rostock, Rostock, Germany; 2 Chair of Technical Dynamics, University of Rostock, Rostock, Germany; 3 Department of Orthopaedics, Traumatology and Hand Surgery, Klinikum Südstadt Rostock, Rostock, Germany; 4 Shiley Center for Orthopaedic Research and Education at Scripps Clinic, La Jolla, CA USA

**Keywords:** Computational models, Musculoskeletal system, Biomedical engineering

## Abstract

The complicated interplay of total knee replacement (TKR) positioning and patient-specific soft tissue conditions still causes a considerable number of unsatisfactory outcomes. Therefore, we deployed a robot-assisted test method, in which a six-axis robot moved and loaded a bicondylar cruciate-retaining (CR)-TKR in a virtual lower extremity emulated by a musculoskeletal multibody model. This enabled us to systematically analyse the impact of the posterior cruciate ligament (PCL), tibial slope, and tibial component rotation on TKR function while considering the physical implant components and physiological-like conditions during dynamic motions. The PCL resection yielded a decrease of femoral rollback by 4.5 mm and a reduction of tibiofemoral contact force by 50 N. A reduced tibial slope led to an increase of tibiofemoral contact force by about 170 N and a decrease of femoral rollback up to 1.7 mm. Although a higher tibial slope reduced the contact force, excessive tibial slopes should be avoided to prevent joint instability. Contrary to an external rotation of the tibial component, an internal rotation clearly increased the contact force and lateral femoral rollback. Our data contribute to improved understanding the biomechanics of TKRs and show the capabilities of the robot-assisted test method based on a musculoskeletal multibody model as a preoperative planning tool.

## Introduction

Total knee replacement (TKR) has become a common surgical procedure to restore knee function in cases such as progressive osteoarthritis. Despite the progressively increasing number of TKR surgeries in the past decades^1,2^, the 20%^3^ rate of unsatisfactory postoperative outcomes constitute a prevalent problem^2^. TKR instability, implant component wear, aseptic loosening, anterior knee pain, among others, are the major reasons for patient dissatisfaction, TKR failure, and revision surgery^4,5^. Studies have shown that the percentage of failure due to instability, possibly leading to TKR revision^6^, after two years of the procedure is 21–25%^4,5,7^. Further studies have reported that 12–20% of TKR revisions are caused by the malalignment or malpositioning of the implant components, both linked to decreased implant survival^8,9^ and unsatisfactory patient outcomes^9–12^. These numbers emphasise the impact of TKR instability and TKR component malalignment on postoperative outcomes, as these are frequent causes for revision surgeries and patient dissatisfaction. Moreover, it has been shown that even computer navigation is ineffective in accurately controlling axial implant component alignment^13,14^, especially for the tibial component^13^. For improving clinical results and patient satisfaction, it is essential to understand the effects of implant malalignment on the biomechanical behaviour of different total knee endprostheses under the consideration of the patient-specific musculoskeletal system, i.e., especially the condition of soft tissue structures affecting joint function.

Concerning the posterior cruciate ligament (PCL), Verra *et al*.^15^ have described that to date, there is no consensus concerning the retaining or sacrificing of the PCL, which is opposed to the findings of other studies that highlight the importance of the PCL for restoring tibiofemoral kinematics^16–20^. Regarding TKR component malalignment, recent studies have revealed that the acceptable range of the tibial slope is still controversial^18,19,21,22^, although evidently important for postoperative outcomes. Increasing the tibial slope can result in posterior wear of the tibial insert and may lead to knee instability^21,23^ but has also been shown to improve knee flexion and reduce quadriceps force for knee motion^21,24^. The definition of an appropriate intraoperative rotational positioning of the femoral and tibial components is still a controversial topic^12,16,25–29^, although crucially important for functional and postoperative outcomes^27^ as well as for the longevity of TKRs^11^. Component malalignment has been shown to affect postoperative knee kinematics and to contribute to postoperative pain, implant component wear or loosening, and to instability as well as increased tibiofemoral pressure^9,12,16,28–32^. Despite these studies delivering valuable insights into the biomechanical behaviour of TKRs, the examination of isolated parameters like TKR component malpositioning or soft tissue damage and the comprehensive analysis of their complex interplay as the surgical parameters of knee joint dynamics have not yet been achieved so far.

During surgery, clinicians are only able to subjectively examine the knee joint by analysing the flexion and extension gap, however, without the capability to quantitatively evaluate neither knee kinematics nor kinetics^33^. Furthermore, *in-vitro* studies lack the ability to analyse the impact of isolated influencing factors on the overall musculoskeletal dynamics in terms of reproducibility, and most of these studies do not consider the physiological soft tissue response during dynamic activities^33–37^. Such *in-vivo* measurements are ethically not possible. On the other hand, computational studies^18,25,36,38^ lack the capability to effectively simulate realistic contact properties.

Therefore, we established a robot-assisted test method, in which a six-axis robot actuated a commercially available bicondylar cruciate-retaining (CR)-TKR within a virtual lower extremity emulated by a musculoskeletal multibody simulation model. In the hardware-in-the-loop (HiL) simulation approach^39,40^, the robot loads and moves the TKR components according to the motion angles and reaction forces/torques computed by the musculoskeletal multibody model, while the measured positions and loading of the TKR components are fed back into the model. This combination of a musculoskeletal multibody model with an industrial six-axis robot allows for the integration of realistic contact boundary conditions resembling the virtual implantation of the TKR into the musculoskeletal multibody model, thereby, mitigating the limitations of solely experimental or numerical studies. Thus, the HiL simulation enables the systematic analysis of the impact of the PCL, tibial slope, and tibial component rotation on the function of the TKR under the consideration of not only real, physical TKR components but also physiological-like soft tissue forces during dynamic motions.

Consequently, we hypothesise that the HiL simulation approach is capable of predicting realistic knee joint dynamics under the representation of realistic contact mechanics and physiological-like soft tissue representation, thereby, allowing the comprehensive analysis of surgical parameters and their effects on the TKR joint dynamics of bicondylar CR-TKRs.

## Materials and Methods

**Figure 1:**
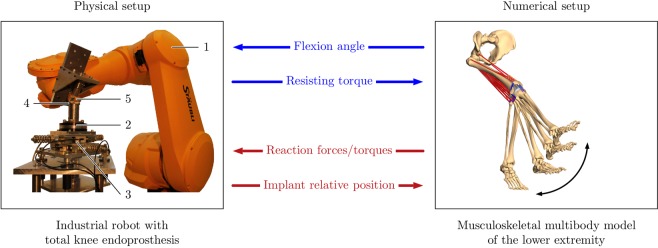
Functional principle of the hardware-in-the-loop simulation to evaluate total knee replacements during dynamic activities. The physical setup (left) consists of an industrial robot (1) with a six degrees of freedom force-torque sensor (2), mounted on a compliant support (3) with the tibial component (4). The end-effector is equipped with the femoral component (5). The robot moves and loads the total knee endoprosthesis according to the flexion angle delivered by the musculoskeletal multibody model of the lower right extremity, which constitutes the numerical setup (right). The musculoskeletal multibody model includes bones, muscles, ligaments, and a virtually implanted total knee endoprosthesis. Motion sequence and reaction forces/torques are transferred to the robot, closing the loop.

A detailed description of the HiL setup for TKR testing as well as the validation of the used control strategy is provided in the study of Herrmann *et al*.^39^. In line with this work, the HiL setup consisted of both a physical and numerical setup (Fig. 1). Hybrid position-force control was necessary for one unconstrained rotational direction (flexion/extension) and five constrained directions (adduction/abduction, internal/external rotation, anterior-posterior translation, superior-inferior translation, and medial-lateral translation). The bidirectional communication between industrial robot, sensors, and musculoskeletal multibody model was realised by embedding the components involved in a control system^39,40^.

The physical setup composed of a six degrees of freedom industrial robot (TX200, Stäubli Tec-Systems GmbH, Bayreuth, Germany) combined with a six degrees of freedom force-torque sensor (Omega 160, ATI Industrial Automation, Apex, NC). The force-torque sensor and tibial component were attached to a ground-fixed compliant support with translational displacement sensors (MSK 5000, SIKO, Buchenbach, Germany). The femoral component was attached to the end-effector. The tibial component, as well as the force-torque sensor, was physically located in such a way that the vertical axis was aligned parallelly to the tibial plateau’s surface normal and the sensor’s axis of maximal load, respectively (Fig. 1). The Multigen Plus Knee System (Lima Corporate, Villanova di San Daniele del Friuli, Italy) representing an unconstrained bicondylar PCL retaining multi-radius design was used for all investigations. The femoral component (size #3) had a symmetric design and was composed of cobalt–chromium alloy (Co28Cr6Mo). The tibial component (size #3) was composed of titanium alloy (TiAl6V4). For the HiL simulations, a fixed-bearing ultra-high molecular weight polyethylene liner (10 mm) was used. A physical patella component was not used as the patellofemoral joint was not physically considered on the robot but in the musculoskeletal model. Relative displacements of the implant components were measured via the end-effector’s position sensor and the support’s displacement sensors.

Briefly, in the first control loop associated to the unconstrained direction of flexion/extension, the robot moved the femoral component according to the desired flexion angle under position control, which was obtained from the musculoskeletal multibody model. An occurring resisting torque due to the friction of TKR component contact during motion was measured and fed back to the musculoskeletal multibody model, closing the first control loop. The remaining five degrees of freedom was force-torque controlled in such a way that the TKR component contact loads were in equilibrium with the resulting loads calculated by the musculoskeletal multibody model. These reaction forces and torques were applied to the physical TKR by shifting and rotating the femoral component in the constrained directions. Thus, the second control loop was closed by transferring the modified relative displacements and rotations back to the musculoskeletal model. In this way, two control loops were defined by handling the two complementary sets of spatial movement directions.

A subject-specific musculoskeletal multibody model of the lower extremity was generated using the multibody software SIMPACK (V9.6, Dassault Systèmes Deutschland GmbH, Gilching, Germany), to emulate the anatomical environment of TKR in the HiL simulation. First, a model of the native knee joint was built and validated. To incorporate the model into the HiL simulation, according to Fig. 1, the same TKR used in the physical setup was virtually implanted. Different surgical and implant specific parameters pertaining to TKR dynamics were then investigated by the HiL simulations. The musculoskeletal model was derived from the lower right extremity (as approved by the ethical review committee of the University of Rostock, register number A 2014-0146, Rostock, Germany) of an alcohol-fixed male cadaver specimen (age 74 years, height 176 cm, weight 80 kg), reconstructed from a CT/MRI dataset in AMIRA (v.5.4.1, FEI Company, Hillsboro, OR, USA) according to the workflow described by Kluess *et al*.^41^. The experiment and methods were performed in accordance with relevant guidelines and regulations. Informed consent for study participation was obtained from the donor. The study was approved by the local ethics committees (ethical review committee of the University of Rostock, register number A 2014-0146, Rostock, Germany). The 3D surface models of bones and ligaments were imported into GEOMAGIC studio (v.13, 3D Systems, Rock Hill, SC, USA) to correct and smoothen surfaces. The musculoskeletal model mimicked the clinically relevant load case of a passive knee flexion/extension^42^ in a seated position without contact of the foot to the ground, as shown in Fig. 1. Thereby, the flexion angle was kinematically prescribed. Accordingly, an open kinematic chain was built up that starts at the pelvis, and femur was assumed to be fixed and continue with the patella, tibia and fibula as rigid bodies. The ankle joint was kept fixed. The bone and soft tissue masses of each body part were defined as functions of patient body weight using regression equations^43^. The patellofemoral and tibiofemoral joints were implemented with six degrees of freedom by means of a polygon-contact model^44^. In particular, the patellofemoral joint was modelled only *in*-*silico* according to Tischer *et al*.^45^ by a one degree of freedom custom joint describing the path of patella along a femur-fixed path with an arc length, identified from a previous simulation with a polygon-contact model^44^.

Passive ligament and muscle structures were incorporated in the musculoskeletal multibody model as force elements spanning between the anatomical attachment points, which were divided into fibre bundles. The ligament structures included two bundles of anterior and posterior cruciate ligament (PCL), three bundles of medial collateral ligament (MCL), three bundles of lateral collateral ligament (LCL), one bundle of oblique posterior medial collateral ligament (opMCL), two bundles of deep medial collateral ligament (dMCL), two bundles of oblique popliteal ligament (OPL), one bundle of arcuate popliteal ligament (APL) as well as two bundles of posterior capsule (pCAP). The stability of the patellofemoral joint was ensured by three bundles of lateral patellofemoral ligament (LPFL), three bundles medial patellofemoral ligament (MPFL), and three bundles patellar ligament (PL). The ligaments were modelled as one-dimensional nonlinear force elements. The mechanical properties (stiffness, reference length) were taken from the literature (Supplementary Table S1)^46,47^. The force generated by ligament structures followed a nonlinear elastic characteristic with a slack region. Ligament resection was modelled by deactivating the respective force elements in the musculoskeletal model. During the passive deep knee flexion under consideration, the four components of M. quadriceps femoris (M. rectus femoris, M. vastus medialis, M. vastus lateralis, and M. vastus intermedius) were modelled with a constant overall tensile force of 120 N^36^. The deflection of the patella tendon by the trochlear groove was modelled by defining the contact between the trochlear groove and ellipsoid bodies embedded into the quadriceps tendon, enabling wrapping of the M. quadriceps femoris. The musculoskeletal model of the native knee joint was previously validated against experimental data^48^.

### Parameter study on implant position and tibial slope as well as soft tissue condition

The femoral and tibial condyles were virtually resected using a digital model of the native knee joint. Computer-aided design models of the implant components were positioned with respect to the corresponding osseous reference frames of the musculoskeletal model defined by anatomical landmarks, according to Wu *et al*.^49^. Both implant components were aligned with respect to the mechanical axes of the corresponding bones with the help of an experienced orthopaedic surgeon. The anterior cruciate ligament was sacrificed during the virtual implantation, owing to the general practice of its removal during the implantation of a bicondylar CR-TKR. The musculoskeletal model of the lower right extremity with implanted CR-TKR obtained by these procedures was defined as the reference configuration for the subsequent parameter study.

**Figure 2:**
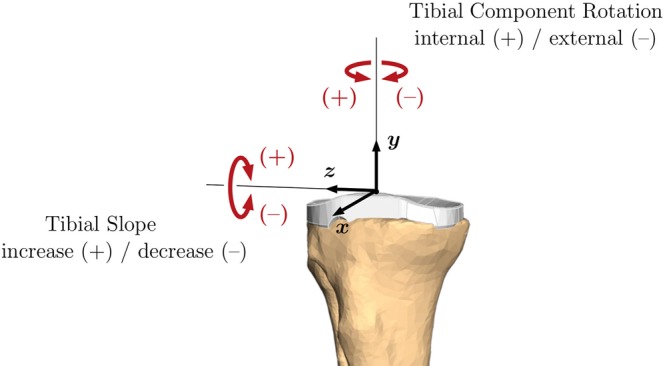
Directions of tibial implant position variations. The tibial implant is rotated relative to its reference configuration with implant axes x, y, z. The reference configuration is defined by an experienced orthopaedic surgeon.

The parameter study summarised in Table 1 analysed the influence of the tibial implant position, as shown in Fig. 2, and ligament conditions during the considered load case of the seated knee flexion and extension. In particular, the flexion movement from 10° to 120° and extension movement from 60° flexion to 10° hyperextension were considered.

**Table 1:** Test matrix for hardware-in-the-loop simulations.

Configuration	Direction	Value	Movement
Reference			flexion/extension
Tibial component rotation	Internal	+3° +6°	flexion
	External	–3° –6°	flexion
Tibial slope	Decrease	–3°	flexion/extension
	Increase	+3° +6°	flexion/extension flexion
PCL resection			flexion

The parameter values are defined relative to the reference configuration.

The HiL simulations with different parameter settings were validated against experimental data from cadaveric knees with CR-TKR^36^ (Supplementary Fig. S2). The HiL simulation results show a good agreement with the results from Kessler *et al*.^36^. The results were contained in the range of values generated from the experimental data, with the exception of tibial internal/external rotation, which was less pronounced but exhibited the screw-home mechanism in accordance with literature^34^. The screw-home mechanism describes an external rotation of the tibia relative to the femur in the final stage of knee extension movement^50^. Furthermore, a general trend of increasing internal rotation with increased knee flexion angle could be demonstrated. In conclusion, the HiL simulation was capable of reproducing the major trends in the experimental results.

The root mean square error (RMSE) between the reference configuration and changed parameters was calculated to evaluate the influence of parameter variation. Internal/external rotation, femoral rollback, flexion facet centre (FFC) translation^51^, and tibiofemoral contact force were compared along the flexion angle to detect the differences between the kinematic and kinetic curves. Three repetitions were recorded to calculate the mean values and standard deviations. The femoral rollback and internal/external rotation were evaluated according to the established joint coordinate system definitions^49,52^.

## Results

All trials (n = 3 for each parameter setting) within the HiL simulations started at 10° knee flexion. Concerning the reproducibility of the HiL setup, low standard deviations were observed in the measurements (less than 0.1° for internal/external rotation, 0.06 mm for femoral rollback, less than 7 N for tibiofemoral contact force). An overview of the data pertaining to the variations in surgical parameters of the biomechanical behaviour of a bicondylar total knee endoprosthesis is provided in Supplementary Table S3.

### Impact of posterior cruciate ligament resection on knee joint kinematics and kinetics

**Figure 3:**
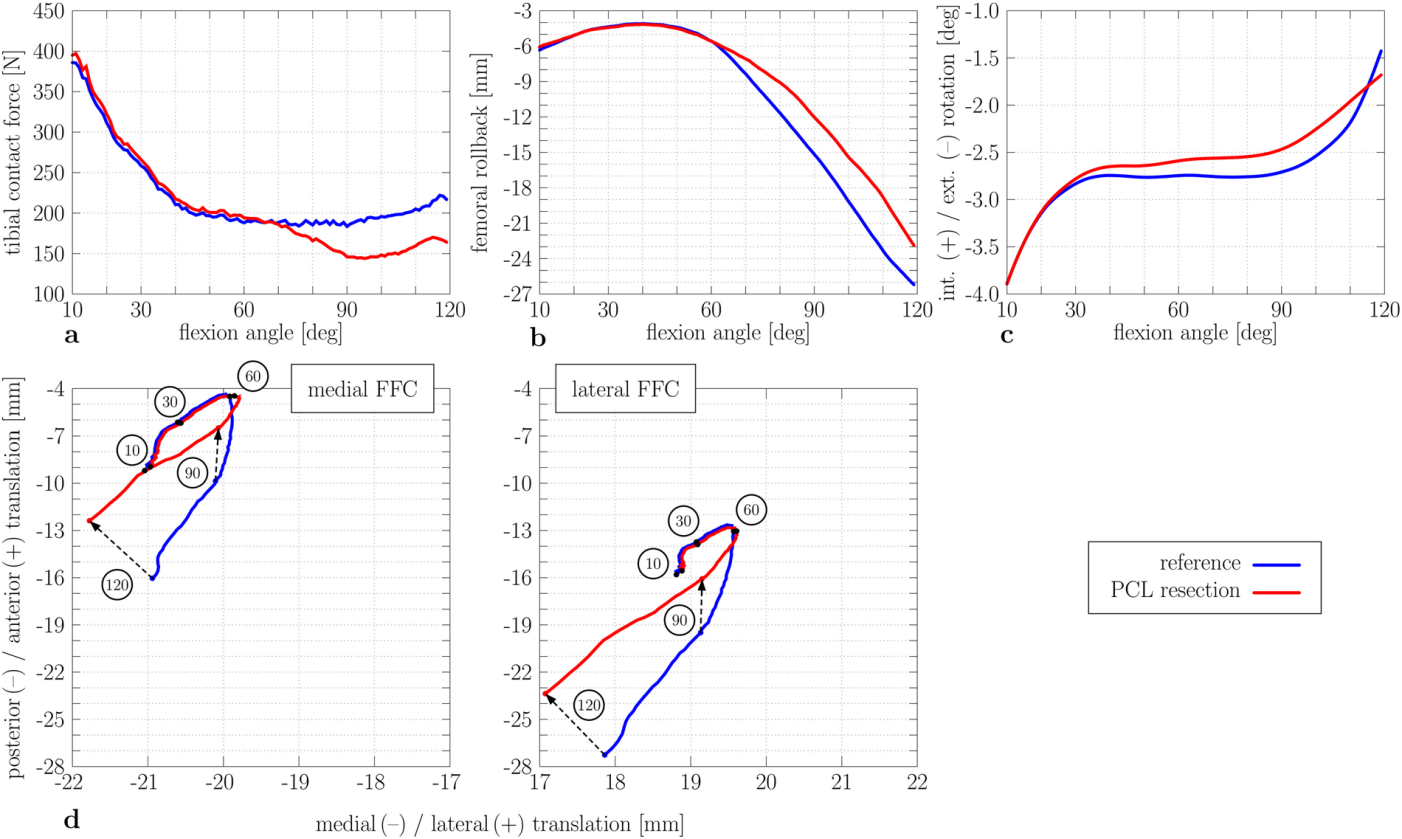
Tibiofemoral kinematics and kinetics results during knee flexion for different conditions of posterior cruciate ligament (reference and resected PCL). Tibiofemoral contact force during knee flexion. (**a**) Femoral rollback (−) during knee flexion. (**b**) Tibial internal (+) and external (−) rotation. (**c**) Flexion facet centre (FFC) position of the medial (left) and lateral (right) femoral component relative to the tibial component and marked flexion angles of 10°, 30°, 60°, 90°, and 120° (**d**) (blue: reference; red: PCL resection).

First, the impact of PCL resection on knee kinematics and kinetics was evaluated (Fig. 3). Generally, the influence of PCL resection can be observed beyond 60° flexion. A decrease in tibiofemoral contact force beyond 50 N (RMSE = 26.58 N) can be detected in deep knee flexion after PCL resection (Fig. 3a). The PCL resection influenced knee kinematics. The femoral rollback shifted more anteriorly (RMSE = 2.3 mm, Fig. 3b), e.g., approximately 4.5 mm during deep knee flexion (110°). The internal and external tibial rotation remained rather unchanged after PCL resection (RMSE = 0.17°), and the screw-home mechanism was not affected by PCL resection (Fig. 3c). Figure 3d depicts the medial and lateral FFC translation of the femoral component relative to the tibial component. After PCL resection, the medial and lateral FFC were positioned at almost similar locations below 60° knee flexion. Paradoxical anterior translation, i.e., the anterior translation of the femur relative to the tibia, was observed for both PCL conditions. With PCL, the paradoxical anterior translation of the medial FFC was limited below 55° knee flexion, whereas the paradoxical anterior translation of the medial FFC continued to approximately 60° of knee flexion after PCL resection.

### Impact of tibial slope on knee joint kinematics and kinetics

**Figure 4:**
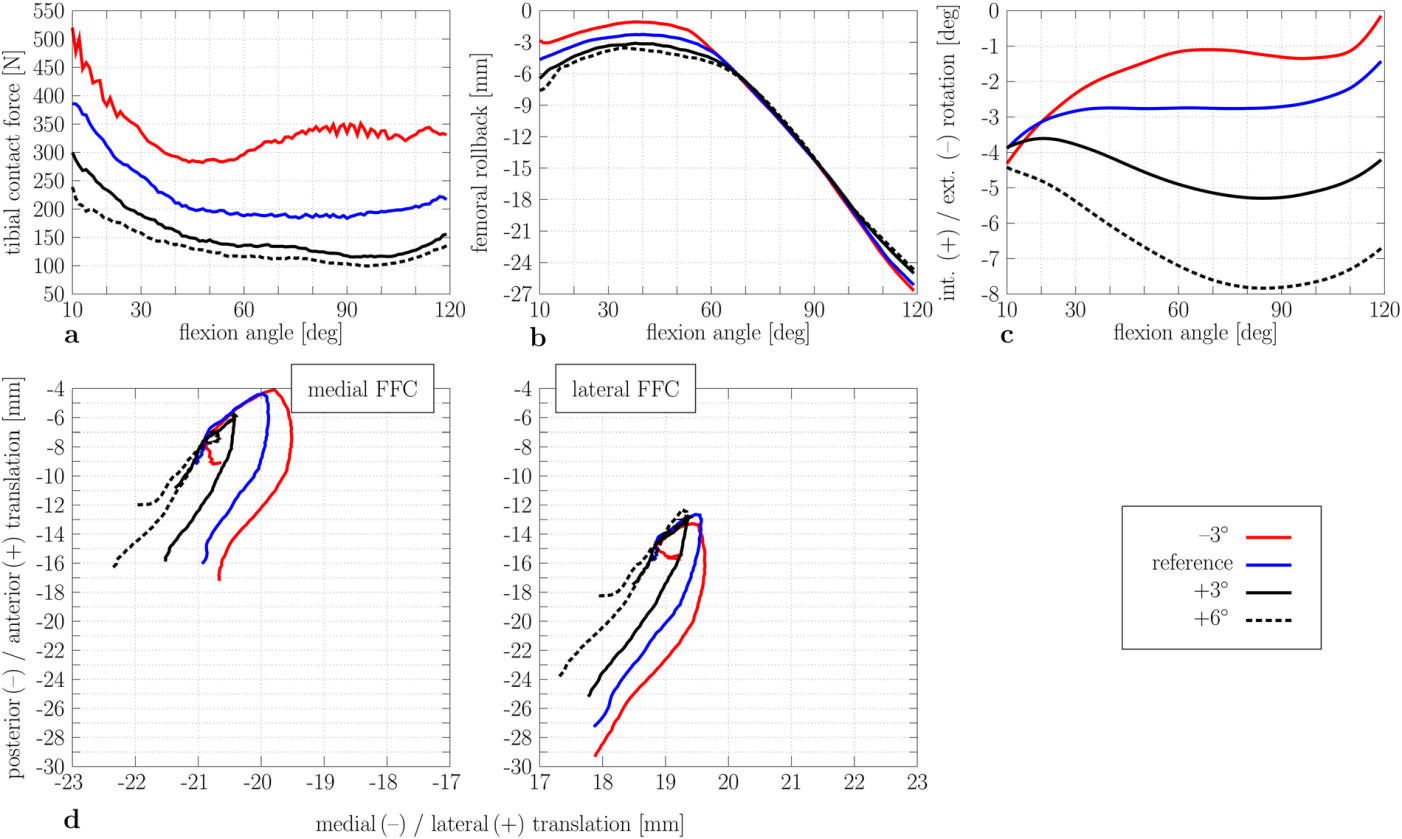
Tibiofemoral kinematics and kinetics results during knee flexion for different tibial slopes (reference, −3°, +3°, and +6° tibial slope). Tibiofemoral contact force during knee flexion. (**a**) Femoral rollback (−) during knee flexion. (**b**) Tibial internal (+) and external (−) rotation. (**c**) Flexion facet centre (FFC) position of the medial (left) and lateral (right) femoral component relative to the tibial component (**d**) (+: increase of the tibial slope; −: decrease of the tibial slope).

The impact of tibial slope on tibiofemoral kinematics and kinetics in CR-TKR is depicted in Fig. 4. The tibiofemoral contact force increased over the whole range of flexion angles due to a decreased tibial slope of −3°, e.g., at 80° flexion by up to 170 N (RMSE = 117.9 N, Fig. 4a). Conversely, an increased tibial slope reduced the tibiofemoral contact force, e.g., an increased tibial slope of +3° led to a decreased tibiofemoral contact force (RMSE = 67.8 N). However, the impact of decreasing the tibial slope by −3° was much higher, e.g., a + 3° tibial slope relative to −3° led to a decrease in the tibiofemoral contact force by up to 54%. Likewise, a change in the tibial slope also had an impact on the anterior-posterior kinematics of the CR-TKR (Fig. 4b) in the early- to mid-flexion range (10°–70°) but was less distinct for the high-flexion range (70°–120°). A small tibial slope decreased the femoral rollback by up to 1.7 mm at the early- to mid-flexion (10°–70°), while slightly higher posterior femoral translation was detected at higher flexion angles (RMSE = 0.72 mm). In contrast, with a high tibial slope, the femoral component moved more posteriorly from the early- to mid-flexion, and the component moved slightly more anteriorly from 90° to 120° knee flexion, reducing the amount of anterior-posterior translation (RMSE = 0.72 mm). Concerning tibial internal/external rotation (Fig. 4c), an increase of tibial slope led to more external tibial rotation (RMSE = 2.1°), and the decrease led to a more internal tibial rotation (RMSE = 1.2°). Compared to the reference configuration, an increased tibial slope (+3°) resulted in an increased shift of the medial and lateral FFC to a more posterior position in the early- to mid-flexion range (Fig. 4d).

### Impact of tibial component rotation on knee joint kinematics and kinetics

**Figure 5:**
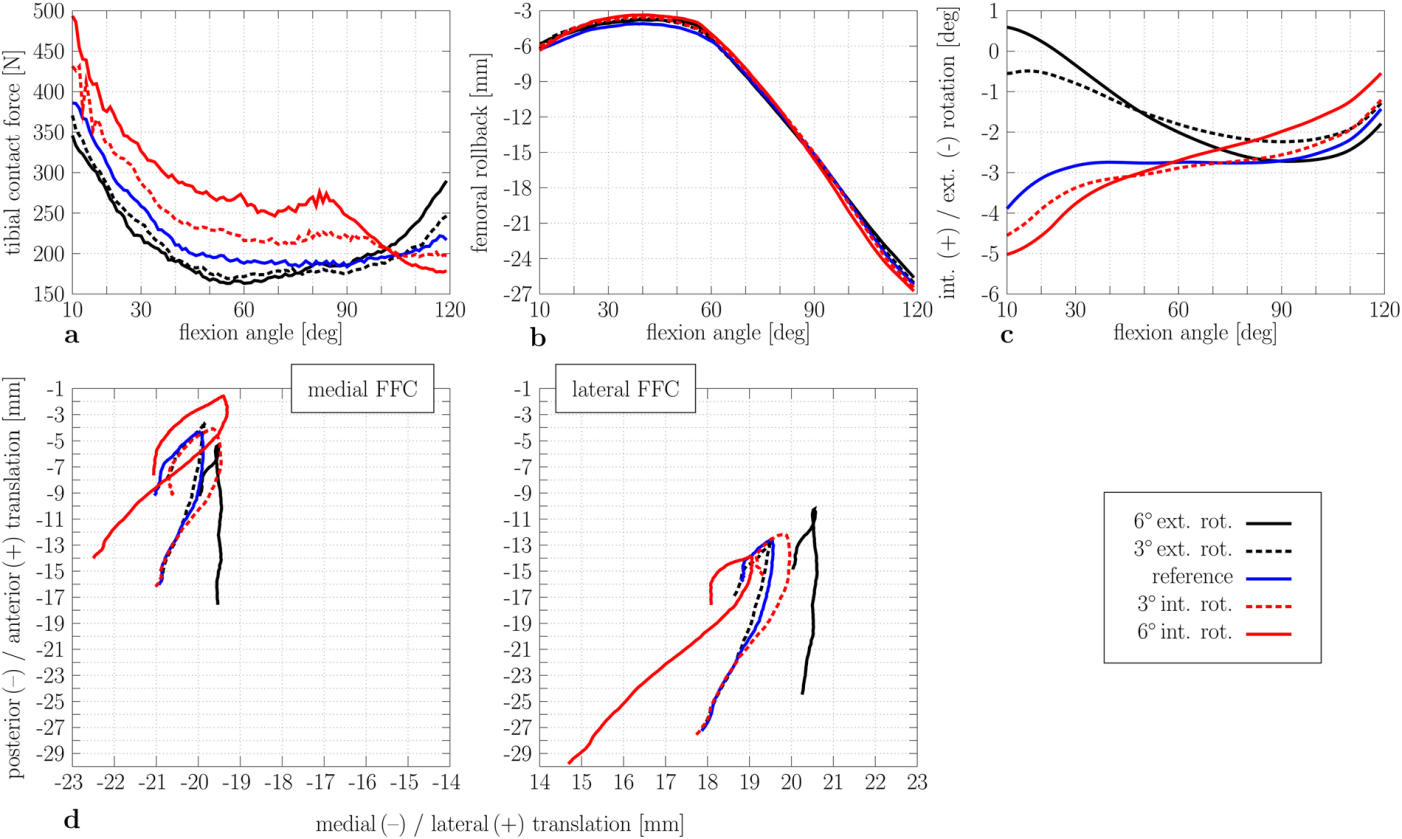
Tibiofemoral kinematics and kinetics results during knee flexion for different tibial component rotations (reference, 3°, and 6° internal/external rotation). Tibiofemoral contact force during knee flexion. (**a**) Femoral rollback (−) during knee flexion. (**b**) Tibial internal (+) and external (−) rotation. (**c**) Flexion facet centre (FFC) position of the medial (left) and lateral (right) femoral component relative to the tibial component (**d**) (int: internal rotation; ext: external rotation).

The internal rotation of the tibial component increased the amount of the tibiofemoral contact force (RMSE = 30.24 N) (Fig. 5a), whereas external rotation decreased the tibiofemoral contact force (RMSE = 18.56 N). Likewise, the rotation of the tibial component also influenced on the anterior-posterior kinematics in CR-TKR (Fig. 5b); e.g., a 3° internal rotation of the tibial component resulted in a slightly higher posterior translation of the femur (RMSE = 0.38 mm). Compared to the reference configuration, a 3° external rotation led to a slightly lower femoral rollback (RMSE = 0.32 mm). Up to 70°–80°, an externally rotated tibial component led to a more internal tibial rotation compared to the reference configuration (Fig. 5c) (RMSE = 1.4°). Internal rotation led to a more external tibia rotation (RMSE = 0.38°), which lasted up to a 60° flexion angle. Then, the course switched to a more internally rotated path. For the reference configuration, the lateral FFC moved more posteriorly compared to the medial FFC (Fig. 5d) due to the increased internal rotation of the tibial component (lateral rollback). A more internally rotated tibial component (+3°) increased the lateral rollback (RMSE_+3°_ = 0.8 mm). The opposite effect can be detected by an external tibial component rotation (RMSE_–3°_ = 0.81 mm).

**Figure 6:**
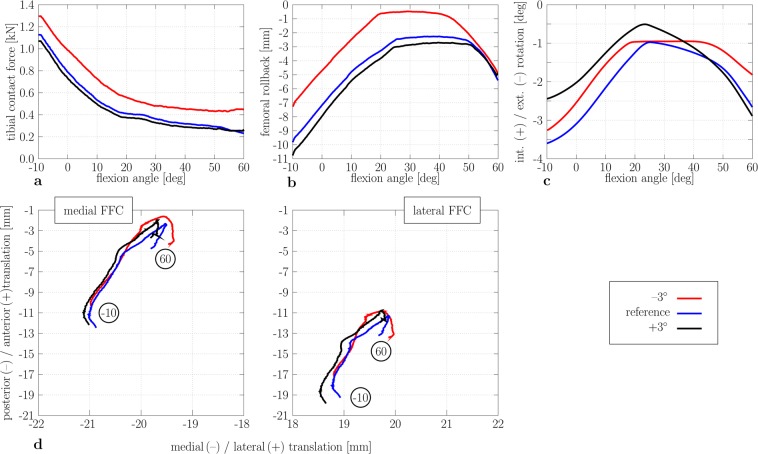
Tibiofemoral kinematics and kinetics results during knee extension for different tibial slopes (reference, −3°, and +3° tibial slope). Tibiofemoral contact force during knee flexion (**a**). Femoral rollback (−) during knee flexion (**b**). Tibial internal (+) and external (−) rotation (**c**). Flexion facet centre (FFC) position of the medial (left) and lateral (right) femoral component relative to the tibial component (**d**) (+: increase of the tibial slope; −: decrease of the tibial slope).

In contrast to the knee flexion movement, the tibiofemoral contact force increased considerably during knee extension up to 1,100 N due to hyperextension (Fig. 6a). This increase could be attributed to the over-tightening of the ligaments. The posterior translation increased with enhanced knee extension angles. Generally, both the screw-home mechanism and the femoral rollback mechanism can be simulated (Fig. 6c,d). The influence of the tibial slope was similar to the knee flexion movement.

## Discussion

The influencing factors on the knee joint biomechanics are difficult to analyse under reproducible conditions for a variety of reasons. Currently, knee joint stability is qualitatively assessed intraoperatively by the surgeon via manual varus-valgus and anterior-posterior stress testing at 0° and 90° knee flexion, while the stability in the mid-flexion range is not quantitatively considered^33^. In addition to intraoperative examinations, knee joint stability can be investigated using computational, experimental, or clinical studies. Although computational studies have shown great potential in clinical decision-making^11,12,17,18,21,38,45,48,53,54^, they are associated with uncertainties regarding the prediction of realistic contact conditions. On the other hand, experimental studies^16,24,26,34,36,55^ lack the consideration of the complex soft tissue structures acting at the lower extremity. Moreover, human cadaver specimens do not allow reproducible and comparable evaluations of different parameters due to the limited acquisition of samples and their decay. Lastly, clinical studies^3,8,10,19,22,28,56–59^ are ethically limited, as extensive parameter studies into implant position and design are not possible.

To overcome these limitations, we deploy a validated HiL setup^39,60^, in which an industrial robot actuates a TKR interacting with a musculoskeletal multibody model of the lower right extremity. Within this setup, we combine the advantages of experimental and numerical methods to analyse the effects of potential surgical errors on the resulting knee joint dynamics in a reproducible musculoskeletal environment. In this manner, we systematically evaluate influencing factors such as implant position, tibial slope, and soft tissue condition on CR-TKR biomechanics during a realistic load case under physiological-like and reproducible conditions. Generally, we have been able to show that both the screw-home and the femoral rollback mechanisms can be demonstrated in CR-TKR in close approximation. In other words, our HiL setup is capable of predicting the typical kinematics patterns^34,51^ of the knee joint under reproducible conditions. Additionally, several clinical phenomena have been observed, e.g., a loss of the physiological screw-home mechanism in agreement with the findings reported by other research groups^26,30,34^. This can be explained by the loss of menisci and anterior cruciate ligament. Furthermore, the used symmetric CR-TKR shows a paradoxical anterior translation of the femur for all variations. Some research groups have attributed paradoxical translation to the loss of anterior cruciate ligament or the femoral design^30^.

Regarding the PCL, the results show that the insufficiency of the PCL structure due to damage, intraoperative resection, or rupture after TKR can be related to anterior translation, e.g., reduction of femoral rollback, lower tibiofemoral contact force, and may lead to unfavourable anterior-posterior instability^17,61^ and enlarged flexion gap^19,62,63^. The findings of our study are in accordance with those of recent studies^17,20^; e.g., a decrease in the tibiofemoral contact force after PCL resection was noted at 90° due to a larger flexion gap. As expected, no differences in kinematics and kinetics were observed in the early flexion angles after PCL resection; e.g., the medial and lateral FFC were positioned at almost similar locations. We can confirm the importance of the PCL during soft-tissue balancing to restrict posterior tibial translation, increase femoral rollback, and contribute to knee joint stability. However, a partial release of the PCL can reduce its over-tightening and may prevent excessive femoral rollback leading to stress concentration and the posterior edge loading of the tibial insert and polyethylene wear^61^.

In line with reported findings^17,18,24,38,64^, an increased tibial slope prevents an excessive load on the implant components during flexion. The relaxing of the collateral ligaments and PCL due to the increased tibial slope is due to the decrease in the distance between the ligament attachments during flexion^64^. Our results show that soft tissue structures are unloaded due to increased tibial slopes, which are also in accordance with reported findings^17,18,24,38^. Increasing tibial slope leads to a higher posterior translation of the femoral component in the early- to mid-flexion range (10°–70°), while slightly higher anterior femoral translation was found at higher flexion angles, thus reducing the amount of anterior-posterior translation, which is in excellent agreement with the cadaver study of Dai *et al*.^22^ for the same load case. Contrarily, further studies^18,21,53,55^ have reported continuous posterior translation due to increased tibial slopes. A higher posterior translation will lead to a greater lever arm of the quadriceps, which can be expected to improve motion efficiency, resulting in a reduced quadriceps force^21,24,53^. This can be explained with the resulting posterior force on the femur produced by an increased tibial slope and the gravity, thus locating the femur more posteriorly due to the ACL loss^58^. For a higher tibial slope (+3°), our results show a posterior translation of the medial FFC compared to a smaller tibial slope (−3°). A similar pattern of results has been obtained in recent studies^57,58^. The key factors in the determination of tibial slope are the native tibial slope of the patient and the TKR design used. Some designs have tibial inserts with a built-in slope, like in our study (+7°). In this case, surgeons should pay attention to aim for smaller tibial slopes, as our results indicate certain complications. Regarding designs without built-in slopes, surgeons could aim for a higher slope in the tibial cut, as tibial slopes that are too small show undesired results in our study, which may lead to postoperative complications^18,19,21,38,64^. The results of our study suggest that an increased tibial slope has a beneficial biomechanical effect. From the clinical point of view, during surgery, an excessive tibial slope (≥+ 6°) should be avoided for the implant design tested with a built-in slope to prevent the progressive opening of the tibiofemoral joint gap during flexion, affecting the postoperative range-of-motion^55^ and knee joint stability^18,21,23,27,38,64^. A tibial slope of +6° leads to a slightly posterior tibiofemoral subluxation of the lateral compartment, which is associated with flexion instability due to a reduction in collateral and posterior cruciate ligament tension and loosening in mid-flexion, as demonstrated in clinical observations^65,66^.

Concerning the rotation of the tibial component, internal rotation increases the amount of the tibiofemoral contact force and vice versa, which is in agreement with Kuriyama *et al*.^12^. Compared to the reference configuration, an externally rotated tibial component leads to a slightly lower femoral rollback, whereas an opposite effect is seen in rotating the tibial component more internally, which are in accordance with Steinbrück *et al*.^16^. Furthermore, an externally rotated tibial component shifts the medial FFC more posteriorly in accordance with Thompson *et al*.^11^, which can explain the paradoxical anterior translation that has been observed after TKR^30^. We conclude that excessive internal rotation of the tibial component should be avoided, which is in agreement with clinical observations^9,16,27–29,31,32^. On the other hand, it seems that a 3° external rotation of the tibial component at the maximum does not have a negative impact. These findings are clinically valuable because of the difficulty of clinically assessing axial tibial component rotation even in surgery using computer navigation, where an error in tibial component internal rotation averages 5 to 7 degrees^13,14^. Within our study, the influence of such rotation on the dynamics of CR-TKR has been evaluated.

The presented findings confirm our hypothesis that varying surgical parameters influence the knee joint dynamics of a bicondylar CR-TKR. Even small surgical errors that may not be accurately detectable with intraoperatively used tools^13,14^ can lead to considerable differences in the tibiofemoral contact forces and kinematics. However, our present study has some limitations. Since the HiL setup is a control loop, the operational stability of the setup influences the simulation results, as verified in a previous work^39^. The computational model has limitations in terms of the idealisations and simplifications of the biomechanical system promoted by the multibody approach, which approximates the system dynamics through rigid bodies, geometric constraints, and discrete force elements. Also, active muscle forces have not been included because the loads used in this study represent those applied during examination in which the knee is passively flexed by a surgeon^22,33^. Furthermore, we have implemented a clinically relevant load case of the knee extension to show the enormous feasibility of the test setup. The predicted tibiofemoral contact force is in agreement with the measured *in-vivo* tibiofemoral contact force^36^, and the transfer to an active load case can be realised^42^.

The wrapping of the ligament structures has not been considered, but it is currently being conducted in studies for a more realistic representation of curved ligament and muscle paths. Nevertheless, in this study, the components of the M. quadriceps femoris are considered with ellipsoid structures representing the deflection of the muscle units. In this work, we have focused on the tibiofemoral joint, although the rotation of implant components impacts the patellofemoral joint as well^16^. In further studies, we intend to focus on the patellofemoral joint biomechanics, whereby the generated musculoskeletal model in this work provides the groundwork for achieving these goals. The differences between other studies and the present study can be due to the unavoidable variations in the numeric and cadaveric models, the implant design, the amount of rotation applied, or the investigated load case. For instance, the musculoskeletal multibody model represents only one subject and not anatomical variations and gender changes. Therefore, the results have to be interpreted carefully, as they can be subject-specific^22,25^. Finally, the results can be design-specific and may not be generalisable to all CR-TKR designs, as we used a single implant comprising a multi-radius femoral component and tibial component with a built-in slope of +7°. In further studies, we have to compare different implant designs with different tibiofemoral conformity, as they influence knee joint dynamics and can lead to different results^11^. These differences have to be kept in mind while comparing our data with that from literature.

Despite these limitations, the findings of our study are valuable regarding the clinical information for surgeons to consider for future investigations in CR-TKR and to gain more knowledge into the factors affecting knee joint dynamics. A major strength of our validated HiL approach lies in the combination of the advantages of numerical and experimental methods. That enables us to modify ligament properties, e.g., evaluate the effect of soft tissue release on total knee biomechanics. This approach also includes the mechanical properties of the musculoskeletal apparatus and considers physical contact properties. We can incorporate further TKR designs such as those that allow the comparison of single-radius versus multi-radius as well as fixed versus mobile bearing designs or posterior stabilized TKRs. Currently, the application of the HiL simulation to further load cases and implant designs is in progress as this setup is an important step toward determining more realistic test conditions under reproducible and physiological-like conditions.

In summary, a commercial CR-TKR with varying implant positions (rotation of the tibial component) and tibial slopes as well as soft tissue conditions (PCL intact versus resected) was used in the HiL simulations. The clinical relevance of our results suggests that a CR-TKR that enables tight ligament tensions may provide the opportunity for surgeons to add more tibial slope to relieve them. The results of the present study suggest that a tibial slope of more than +6° poses a risk of inducing flexion instability. Our results support the clinical finding that the internal rotation of the tibial component is more critical than the external rotation and the PCL resection. In addition, as CR-TKR kinematics and kinetics are affected by the degree of the tibial slope, surgeons should be careful in applying the tibial slope in CR-TKR owing to it being a critical factor securing desirable postoperative results. We have been able to show that any variation in surgical parameters as well as soft tissue structures affects musculoskeletal dynamics, particularly for instability scenarios. Using the presented HiL simulation, we have shown that the investigated parameters can influence the longevity of TKRs, e.g., unnatural movement of implant components (screw-home) and knee joint instability leading to material damage and may contribute to premature wear. In conclusion, our presented approach may improve the understanding of the influence of clinically relevant parameters, such as ligament insufficiency and implant position, on the biomechanical behaviour of TKRs under reproducible and physiological-like test conditions.

## Supplementary information

Dataset 1
