# Supplementary material for: Effect of surgical parameters on the biomechanical behaviour of bicondylar total knee endoprostheses – A robot-assisted test method based on a musculoskeletal model
Source: Sci Rep. 2019 Oct 10;9:14504. doi: 10.1038/s41598-019-50399-3 (PMC6787084; doi:10.1038/s41598-019-50399-3)
Supplement: Supplementary file 1 — Dataset 1 [file 41598_2019_50399_MOESM1_ESM.pdf]

Effect of surgical parameters on the biomechanical behaviour of bicondylar total knee endoprostheses – A robot-assisted test method based on a musculoskeletal model

Authors:

Kebbach M. [1], Grawe R. [2], Geier A. [1], Winter E. [2],

Bergschmidt P. [1,3], Kluess D. [1], D´Lima D. [4], Woernle C. [2], Bader R. [1]

[1] Department of Orthopaedics, University Medicine Rostock, Germany

[2] Chair of Technical Dynamics, University of Rostock, Germany

[3] Department of Orthopaedics, Traumatology and Hand Surgery, Klinikum Südstadt Rostock, Germany

[4] Shiley Center for Orthopaedic Research and Education at Scripps Clinic, La Jolla, CA, USA

Supplementary information

Table S1: Mechanical properties (stiffness and reference strain) of the used ligaments are adapted from the literature[47,48]. The ligament structures include two bundles of posterior cruciate ligament (PCL), three bundles of lateral collateral ligament (LCL), one bundle of oblique posterior medial collateral ligament (opMCL), two bundles of deep medial collateral ligament (dMCL), two bundles of oblique popliteal ligament (OPL), one bundle of arcuate popliteal ligament (APL), two bundles of posterior capsule (pCAP), three bundles of lateral patellofemoral ligament (LPFL), three bundles of medial patellofemoral ligament (MPFL), and three bundles of patellar ligament (PL). Stiffness is expressed in Newton per unit strain. Reference strains are used to calculate the ligament lengths in an upright standing reference position.

**tibiofemoral**				**patellofemoral**			
**Ligament**	**Bundle**	**Stiffness**	**Ref. strain**	**Ligament**	**Bundle**	**Stiffness**	**Ref. strain**
PCL	a	3234	−0.24	MPFL	s, m, i	470	0.06
	p	1178	−0.03				
MCL	a	2500	0.03	LPFL	p	120	-0.03
					c	120	0.03
					d	120	0.03
	c	2750	0.05	PL	l, c, m	11000	-0.02
	p	2750	0.04				
opMCL		1350	−0.06				
dMCL	a	1500	−0.10				
	p	2000	−0.03				
LCL	a	750	0.014				
	s	1500	0.024				
	p	1875	0.02				
OPL	p, d	662	0.07				
APL		1270	0.05				
pCAP	l, m	3000	0.05				

a	anterior
c	central
d	distal
i	inferior
l	lateral
m	medial/middle
p	posterior/proximal
s	superior

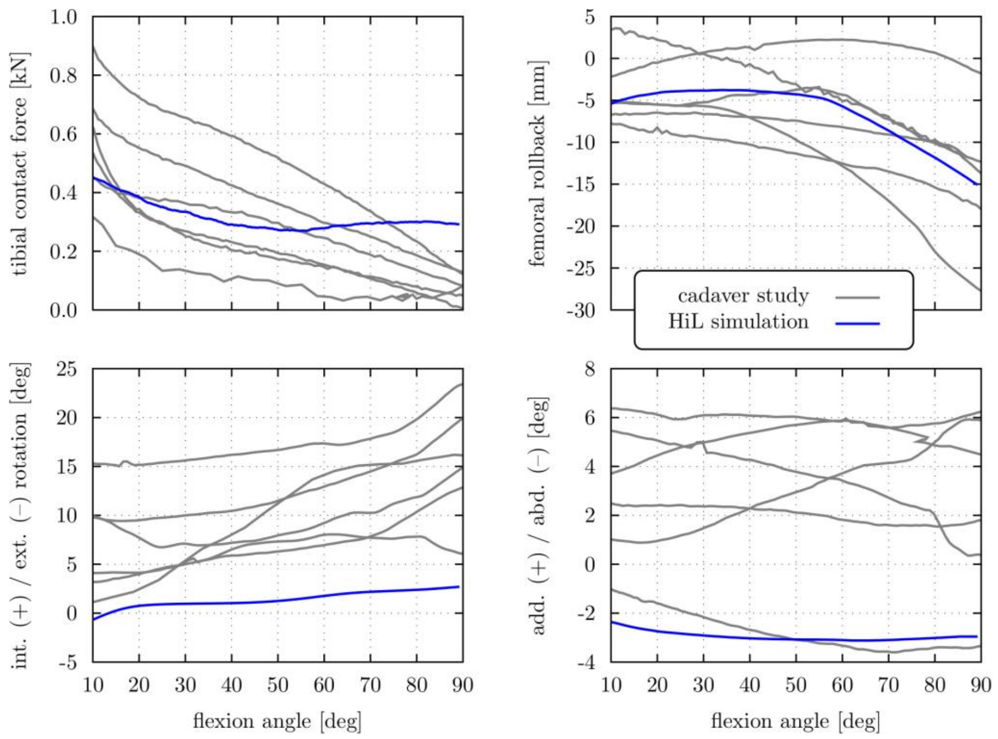

Fig. S2: Hardware-in-the-loop (HiL) simulation validation in terms of kinematics and kinetics. Comparison of hardware-in-the-loop (HiL) simulation outcomes for a seated deep knee flexion (solid blue lines) with respect to human cadaver study by Kessler et al.[37] (solid grey lines). The measurements of the cadaver study were taken from Kessler et al.[37]. Permission is granted to publish under a CC BY open access license.

Table S3: Influences associated with variations in surgical parameters on the biomechanical behaviour of a bicondylar cruciate-retaining total knee endoprosthesis. Impact of posterior cruciate ligament resection, tibial slope, and tibial component rotation on knee joint kinematics and kinetics.

**Configuration**	**Tibiofemoral contact force**	**Femoral Rollback**	**Tibial internal-external rotation**
Posterior cruciate ligament resection	Decreased load beyond 60° knee flexion	Decreased femoral rollback beyond 60° knee flexion	Not affected
**Tibial slope**			
Decrease	Increased load, affecting the knee joint stability	Increased anterior translation in early- to mid-flexion range,	More internal rotation
Increase	Decreased load in the whole flexion range	Increased posterior translation in early- to mid-flexion range	More external rotation
**Tibial component rotation**			
Internal	Increased load in the whole flexion range	Slightly higher posterior translation	More external rotation in early- to mid-flexion range
External	Decreased load in the whole flexion range	Slightly higher anterior translation	More internal rotation in early- to mid-flexion range
